# Transcriptome Analysis Reveals Crosstalk of Responsive Genes to Multiple Abiotic Stresses in Cotton (*Gossypium hirsutum* L.)

**DOI:** 10.1371/journal.pone.0080218

**Published:** 2013-11-05

**Authors:** Ya-Na Zhu, Dong-Qiao Shi, Meng-Bin Ruan, Li-Li Zhang, Zhao-Hong Meng, Jie Liu, Wei-Cai Yang

**Affiliations:** State Key Laboratory of Molecular Developmental Biology, Institute of Genetics and Developmental Biology, Chinese Academy of Sciences, Beijing, China; Wuhan University, China

## Abstract

Abiotic stress is a major environmental factor that limits cotton growth and yield, moreover, this problem has become more and more serious recently, as multiple stresses often occur simultaneously due to the global climate change and environmental pollution. In this study, we sought to identify genes involved in diverse stresses including abscisic acid (ABA), cold, drought, salinity and alkalinity by comparative microarray analysis. Our result showed that 5790, 3067, 5608, 778 and 6148 transcripts, were differentially expressed in cotton seedlings under treatment of ABA (1μM ABA), cold (4°C), drought (200mM mannitol), salinity (200mM NaCl) and alkalinity (pH=11) respectively. Among the induced or suppressed genes, 126 transcripts were shared by all of the five kinds of abiotic stresses, with 64 up-regulated and 62 down-regulated. These common members are grouped as stress signal transduction, transcription factors (TFs), stress response/defense proteins, metabolism, transport facilitation, as well as cell wall/structure, according to the function annotation. We also noticed that large proportion of significant differentially expressed genes specifically regulated in response to different stress. Nine of the common transcripts of multiple stresses were selected for further validation with quantitative real time RT-PCR (qRT-PCR). Furthermore, several well characterized TF families, for example, WRKY, MYB, NAC, AP2/ERF and zinc finger were shown to be involved in different stresses. As an original report using comparative microarray to analyze transcriptome of cotton under five abiotic stresses, valuable information about functional genes and related pathways of anti-stress, and/or stress tolerance in cotton seedlings was unveiled in our result. Besides this, some important common factors were focused for detailed identification and characterization. According to our analysis, it suggested that there was crosstalk of responsive genes or pathways to multiple abiotic or even biotic stresses, in cotton. These candidate genes will be worthy of functional study under diverse stresses.

## Introduction

Abiotic stresses such as drought, cold and high salinity are environmental limiting factors for crop growth and yield. Plants have evolved various biochemical and physiological mechanisms to achieve tolerance and adaption to stresses [1]. Lately, significant progress has been made in identification of stress-inducible genes and components of signaling pathways involved in a variety of abiotic stresses. Many stress-related transcripts and proteins acting in pathways and signaling cascades have been identified, such as the salt-overly-sensitive (SOS) pathway that activated by Ca^2+^ spike from cytoplasm and overcoming the salt damage by maintaining cellular ion homeostasis[2,3], the ICE-CBF /DREB1 pathway critical for the regulation of the cold-responsive transcriptome [4,5], the calcium-dependent protein kinase (CDPK) pathway that taking an important role in hyperosmotic stress response [6], and the mitogen-activated protein kinase (MAPK) pathway essential to both abiotic and biotic stresses [7,8]. 

Various abiotic stresses result in both general and specific effect on plant growth and development [9]. There are multiple pathways involved in stress perception and signaling, some of them are cross-talked at various points [10]. ABA is a major intracellular messenger in the regulation of plant's water status, it is a stress phytohormone that proved to be a player of critical function in signaling network of various stresses, such as drought, cold and salinity [11-13]. Analysis of stress-inducible genes has revealed the existence of ABA-dependent and independent signaling pathways [2,14,15]. In nature, plants often face to several abiotic stresses, rather than a particular one at the same time [10], furthermore, global climatic trends may accentuate this problem [16-18]. Subject to the complex regulatory networks and the crosstalk of various stresses, manipulation of key sensor genes involved in signal transduction or TFs that regulating downstream genes of various pathways, will be an effective way to improve the plant adaption or tolerance to multiple stress conditions [19].

Cotton is one of the most economically important crops worldwide, it is necessary to improve stress tolerance of cotton to increase its yield even under unfriendly environmental conditions. Recently, draft cotton genome sequence was acquired and published, as will benefit gene functional research in cotton [20]. However, in last decade, cotton research is mainly focused on fiber development [21-24], the studies on stress response physiology, identification of novel genes, the function of genes, and stress tolerance improvement with gene engineering have only been attempted more recently, compared with the model plants such as *Arabidopsis*, rice and tobacco [12,25-27], furthermore, most of the studies are engaged in one kind of stress, little is known about the crosstalk of multiple stresses [28-33]. In this paper, we tried to dissect pathways under multiple stresses in cotton with profiling data of global transcription.

So far, transcriptome profiling is an effective and widely used tool to investigate the gene expression dynamics in response to a spectrum of abiotic stresses at the global level in many species [34-36], however, more study on significant crop plants such as cotton is badly needed. In this report, we tend to profile the transcriptome of cotton seedlings stressed with ABA, cold, drought, salinity and alkalinity (pH=11, hereinafter referred to pH in this paper). Differentially expressed genes and related crosstalk network for stress perception, signal transduction and tolerance of cotton were explored using comparative microarray analysis.

## Results and Discussion

### Identification of differentially expressed genes under ABA, cold, drought, salinity or pH stresses

Expression profiling of cotton in response to the abiotic stresses like drought, cold and salinity has been reported recently [29-33], however, most of the studies only focus on one type of stress. In this study, we aimed to identify components of the regulatory network of multiple abiotic stresses. The stress treatments were performed separately on seedlings 4-day after germination (DAG), and RNA was isolated from specimens of 10-day treatment before a 24K Affymetrix cotton genome array [31,37] was conducted to compare the transcription profiles of the plant's response to ABA, cold, drought, salinity and alkalinity (pH=11, hereinafter referred to pH in this paper). The expression level of transcripts was compared, so that genes with more than two-fold change and a p-value of ≤ 0.05 were defined as those with significant different expression. The stress-induced or -repressed genes were identified. 

**Figure 1:**
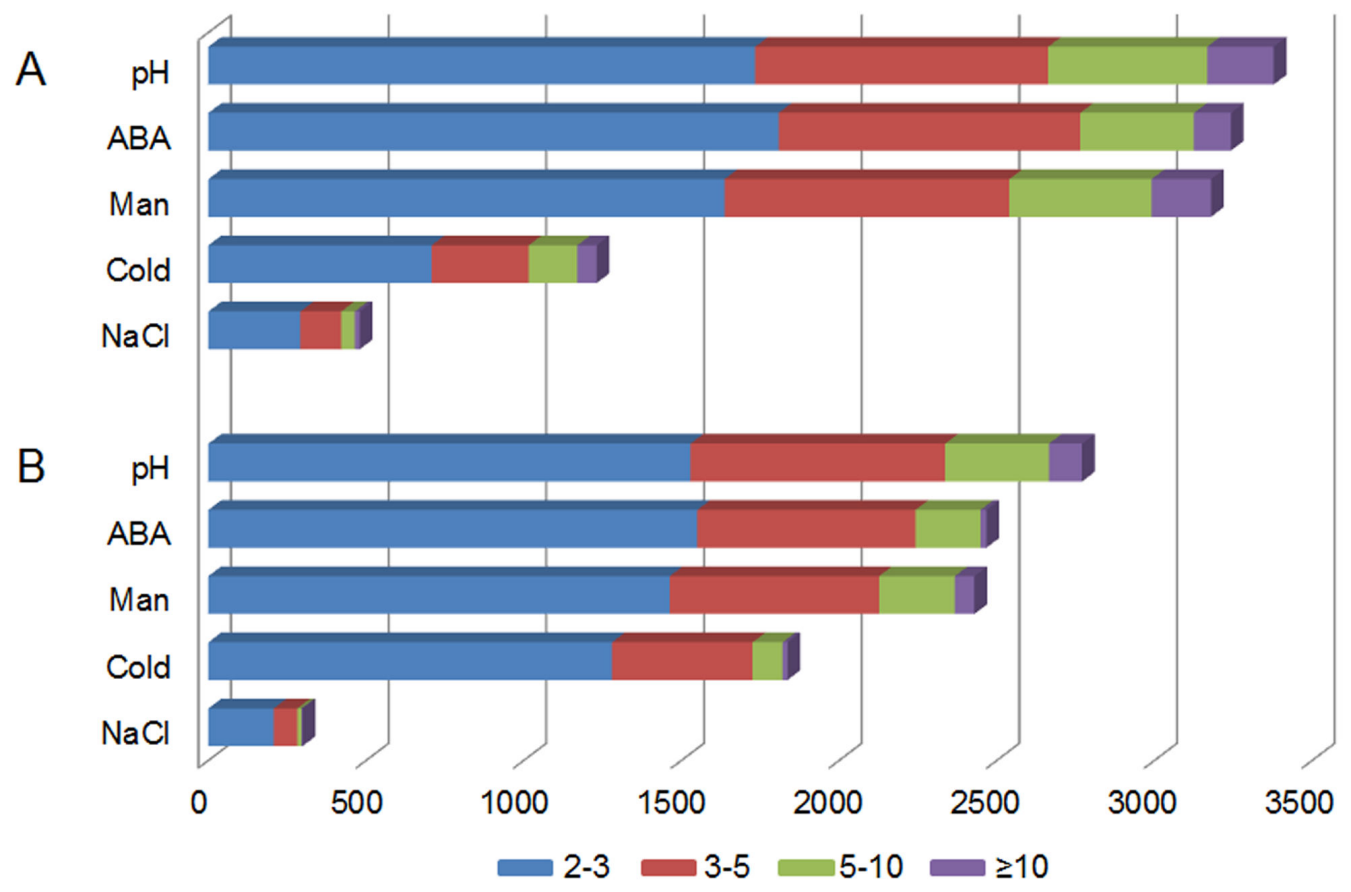
Number and the level of transcripts identified as differentially expressed in cotton seedlings under abiotic stress conditions. The y-axis indicates the treatment of different stress. pH, pH=11; ABA, 1μM ABA; Man, 200mM mannitol; Cold, 4°C; NaCl, 200mM NaCl. The x-axis indicates the number of differentially expressed genes (DEGs), the columns with different color show the fold change of corresponding DEGs. A. up-regulated; B. down-regulated.

As shown in Figure 1, microarray analysis revealed striking difference in gene expression level under ABA (1μM ABA), cold (4°C), drought (200mM mannitol), salinity (200mM NaCl) and pH (pH=11) treatments, which caused 5790, 3067, 5608, 778 and 6148 changed transcripts, respectively. Among these expression changed transcripts, 3242, 1231, 3179, 480 and 3378 genes were identified as stress-induced ones accordingly. There were more induced genes than the repressed in most of the cases except cold treatment (Figure 1-A, B). Most of the significant differentially expressed genes exhibited a 2-3 fold change, and some even showed more than 10-fold change of expression level (Figure 1). We also noticed that there were more than three thousand genes induced by each of ABA, drought and pH stress, while cold and salinity caused expression change of much fewer genes, especially salinity stress, suggesting that there were more genes involved in ABA, drought and pH stress, compared with cold and salinity in cotton seedlings, on the other hand, it is likely because that cotton is a kind of species which is more adaptable to salinity and cold stress compared to other species as *Arabidopsis* [34]. 

In our study, genes with altered expression spanned a wide variety of regulatory and metabolic processes as signal transduction components, TFs, hormone related genes, antioxidants, detoxification enzymes, and biological metabolic processes, a significant number of which were covered in the previous reports of *Arabidopsis*. Several classes of protein kinase and phosphatase involved in abiotic stress signal transduction were up-regulated. It is well-known that MAPK (Mitogen-Activated Protein Kinase) pathways are universal transducers and activated by diverse abiotic stresses, such as cold, salt, heat, drought, wounding, UV irradiation, osmotic shock, ozone or heavy metal intoxication in plants [38]. In our data of microarray, MAPK components are also active under the treatments, a gene encoding MAPK 3 was induced by ABA, cold, drought and pH treatments (Table S1), and it suggested that MAPK signaling pathway was a common way in response of cotton seedlings to multiple abiotic stresses. Previous studies have shown that calcium plays a role as a second messenger during abiotic or biotic stress, when cells are stimulated with external conditions, the concentration of cytosolic free calcium (Ca^2+^) will change accordingly, and calcium-sensing proteins like CDPKs (Calcium-Dependent Protein Kinase), are able to integrate the transient change of Ca^2+^ concentration into a phosphorylation signal [39]. In our results, CPK28, a CDPK member was up-regulated by mannitol and pH treatment but down-regulated by ABA, cold and NaCl. TFs such as DRE (Dehydration-Responsive Element)-binding factor 2 and MYB family members were up-regulated in response to ABA. DRE-binding proteins (DREBs) bind to DRE/C-repeat (DRE/CRT) *cis*-acting element in the promoter region of abiotic stress-responsive genes and activate these genes. Consistently, *GhDREB1A* gene was found induced by cold treatment. A glycolate oxidase (glcD) oxidoreductase gene was activated by NaCl treatment and a *G. hirsutum Gibberellin 20-oxidase 1* (*GA20ox1*) was up-regulated by pH treatment. Interestingly, a gene encoding thiazole biosynthetic enzyme (GeneBank ID: CO076413) was up-regulated over 18 times by all of the five stress conditions, similar result was also reported in other species such as yeast and sunflower [40,41]. Function of more than 40-50% of the identified stress-responsive genes remained unknown. The detail information of above stress-induced and -repressed genes is presented in Table S1.

### Functional classification of the transcripts differentially expressed under various abiotic stresses

**Figure 2:**
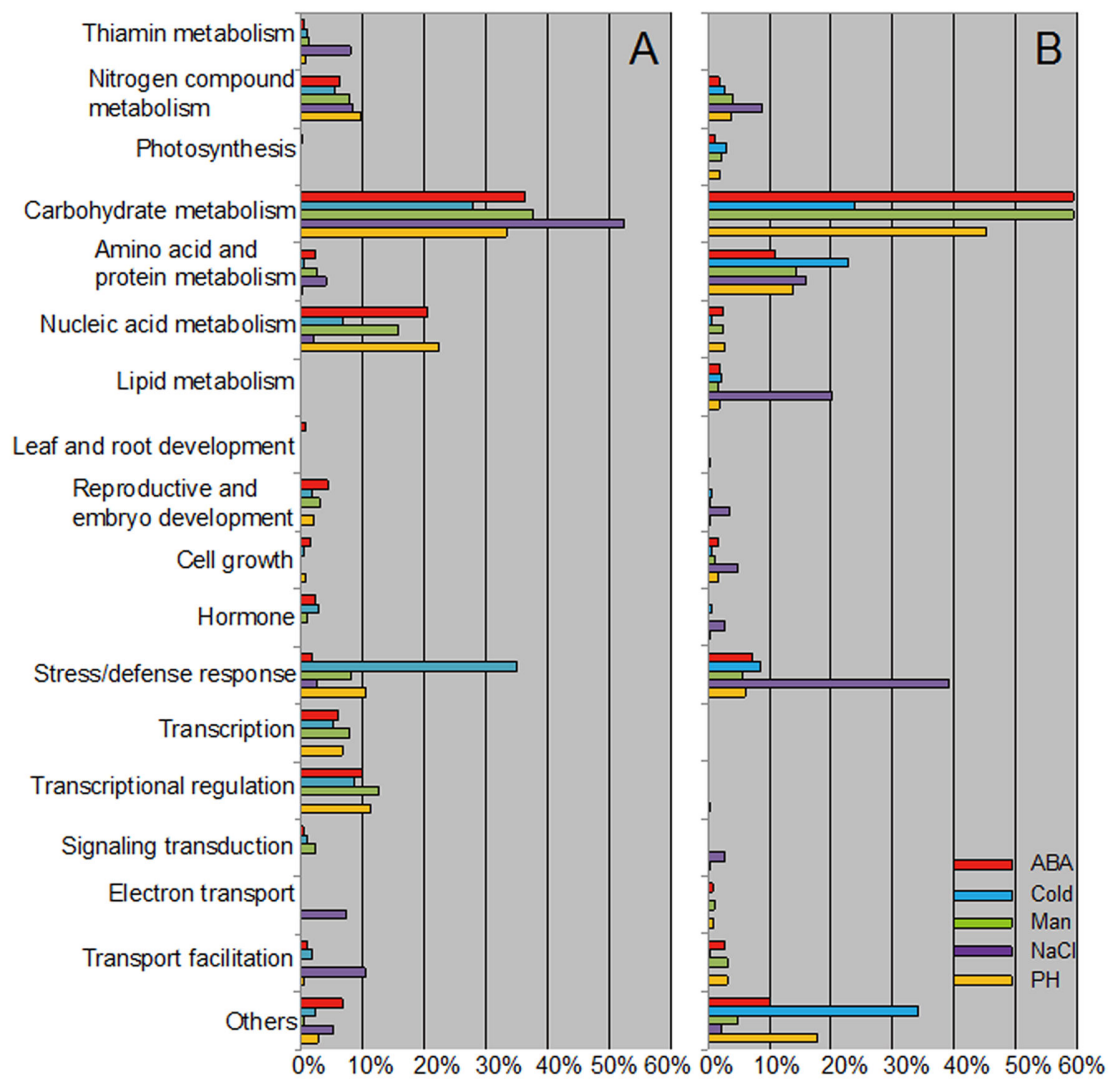
Functional classification of transcripts in cotton seedlings under different abiotic stress conditions. Functional classification of the transcripts was performed using the biological process category of GO Analysis Toolkit and Database for Agricultural Community (http://bioinfo.cau.edu.cn/agriGO/). The x-axis indicates the proportion of the genes expressed in each GO. pH, pH=11; ABA, 1μM ABA; Man, 200mM mannitol; Cold, 4°C; NaCl, 200mM NaCl. The transcripts with fold change greater than 2 or less than 0.5 (only GO term IDs with P ≤ 0.05) were listed. A. up-regulated; B. down-regulated.

 To determine whether the accumulated transcripts were functionally involved in the processes of stress response/defense, we classified the differentially expressed genes according to their putative GO annotation. Since the genes from the 24K Affymetrix cotton genome array were classified into different GO categories, we grouped them into 18 classes according to their percentage of biological process (Figure 2). These categories contained carbohydrate metabolism, stress/defense response, nucleic acid metabolism, amino acid and protein metabolism, nitrogen compound metabolism, transcription, transcriptional regulation, transport facilitation, photosynthesis, thiamin metabolism, lipid metabolism, leaf and root development, reproductive and embryo development, cell growth, hormone, signal transduction, electron transport, and unclassified.

Surprisingly, most of the up- and/or down-regulated transcripts (30-60% of the total) were those involved in the carbohydrate metabolism. Furthermore, many of the up-regulated transcripts were those genes for stress/defense response (10-40%), nucleic acid metabolism (10-20%), or transcriptional regulation (≥10%). Most of the down-regulated transcripts were categorized into amino acid and protein metabolism (10-30%), and stress/defense responses (10-40%). It is worth mentioning that quite a number of transcripts involved in thiamin metabolism were up-regulated while none of these genes was down-regulated. Whereas only down regulation but not up regulation of some photosynthesis genes was found in our data. It suggests that all the abiotic stresses applied in this study can lead to the sudden drop of photosynthesis, and the obvious increase of thiamin as a detoxification factor, as maybe a common mechanism underlying abiotic stress defense of cotton. The detail information of the GO function category of ABA, cold, drought, salinity and pH stress-inducible genes are available in Table S2.

### Crosstalk among the stresses

**Figure 3:**
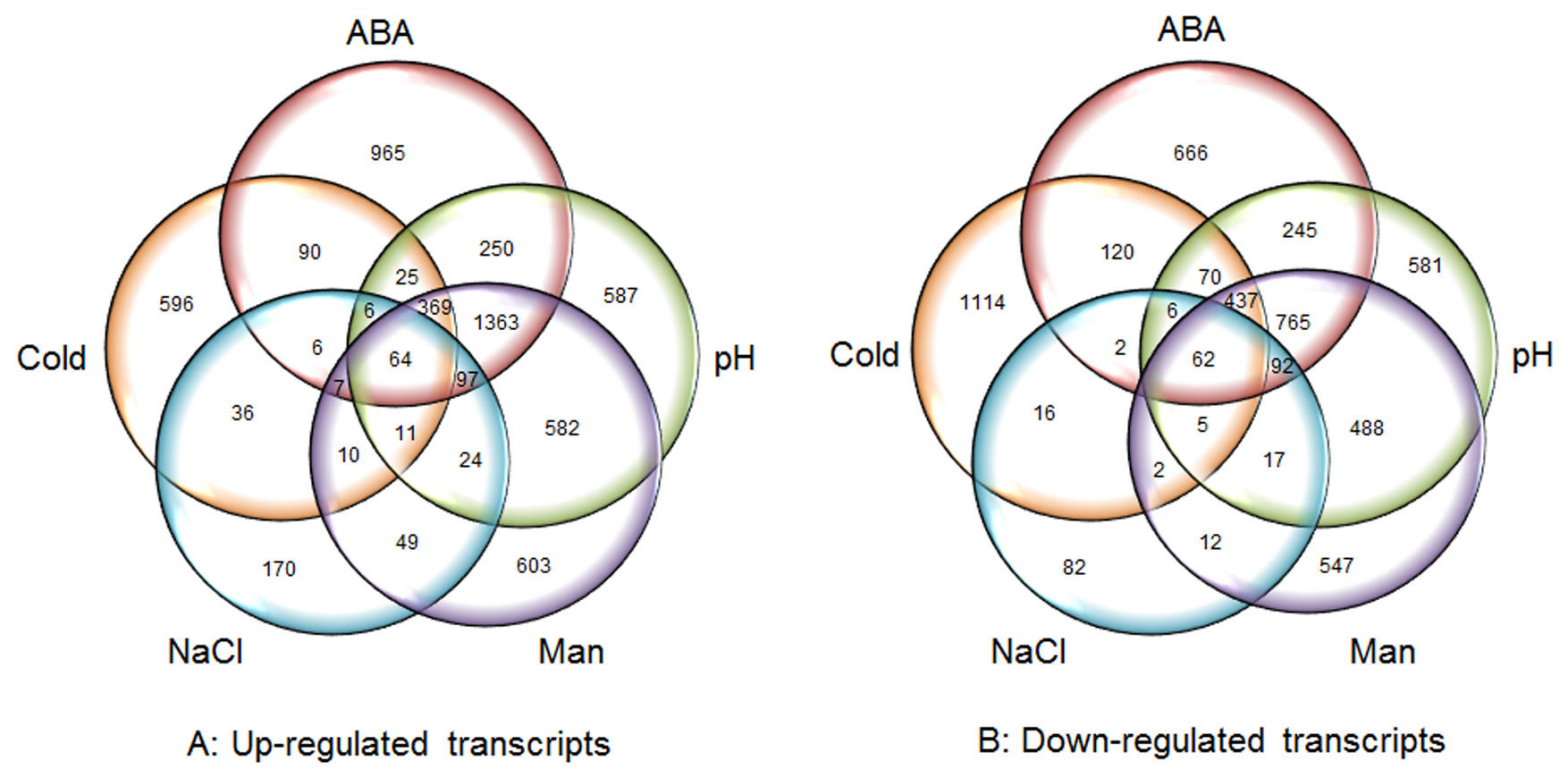
Venn diagram of transcripts identified as up (A) and down (B) regulated in cotton seedings under different abiotic stress conditions. pH, pH=11; ABA, 1μM ABA; Man, 200mM mannitol; Cold, 4°C; NaCl, 200mM NaCl.

In order to identify some common members shared by ABA, cold, drought, salinity and pH, we profiled the transcriptome of cotton seedlings under these conditions. Venn diagrams were constructed and the common differentially expressed genes were identified in all of the five abiotic treatments (Figure 3). Analysis of the overlapping section of the Venn diagram showed that among the up-regulated genes, 1893 were shared in ABA, pH and mannitol treatments, while there were only 83 common up-regulated genes for ABA, cold and NaCl treatments (Figure 3-A). As shown in Figure 3-B, a similar situation occurs in the down-regulated genes, 1356 genes were identified as commonly responsible to ABA, pH and mannitol treatments, however, only 70 common genes showed suppression under ABA, cold and NaCl stresses. The difference between the common members activated or inhibited in above stresses suggested the greater cross-talk among ABA, pH and mannitol than ABA, cold and NaCl. We further classified these common members (Table S3) and the results showed that ABA, mannitol and pH stress shared many components in signaling transduction like MPK3, NADK1 (NAD^+^ kinase 1) as well as GPA1 (G Protein Alpha Subunit 1), furthermore, many transcription factors were also commonly up-regulated such as AP2, MYB, zinc finger and WRKY TF family, while ABA, cold and NaCl did not show any significant common signaling pathway. These results indicated that there was a closer relationship between the drought, alkalinity stress and ABA signal transduction pathways in cotton, while cold and NaCl stress might less efficient in the activation of ABA pathways. Similar results were also reported in previous study on *Arabidopsis* [34]. This was possibly due to the different response to cold, salt and the other stresses in cotton. We also noticed that large proportion of significant differentially expressed genes were specifically regulated under different stresses, this indicated that some special signaling pathways were correspond to certain stress, although the others were cross-talked in many stresses. We identified some molecular components (Figure 3 and Table S3), only a few of these genes had been characterized in cotton or showed homologous in other species like *Arabidopsis* or rice, their function remained largely unknown, so we could not find any pathway or pattern of these genes, it was expected that the function or characterization of these components would be dissected in details with mutants. Among the stresses applied, the most number of genes up- or down-regulated specifically to a stress was 965 or 1114, corresponding to ABA or cold respectively. Sixty-four genes were induced while 62 genes were repressed under all five stresses, and the percentage of these members is low, as 1.97% in ABA, 2.01% in mannitol, 1.89% in pH, 5.2% in cold and 13.3% in NaCl, the detailed information is available as Table S3.

### Characterization of common differentially expressed genes

The common 64 induced and 62 repressed genes were identified and listed (Figure 3, Table S4). In these common genes, 29 induced and 30 repressed genes had been characterized in cotton or showed homologous in other species, like *Arabidopsis* or rice. The differentially expressed transcripts in response to ABA, cold, drought, salinity and pH stress were predicted to encode various functional and regulatory proteins involved in signal transduction, transcription regulation, stress defense/response, secondary metabolism and transport facilitation (Table 1, Table S4). 

**Table 1:** No. of transcripts involved in different functional groups commonly changed under ABA, cold, drought, high-salinity and pH stress.

Putative Function	No.	Description
	**Up-regulated transcripts**	
Rector-like protein kinase	2	alpha-glucan water dikinase 1, alpha-glucan water dikinase 1
Protein phosphatase	2	Protein phosphatase 2C 77, protein phosphatase 2C 12
Phosphorylase	3	ATPHS2, α-glucan phosphorylase isozyme H, starch phosphorylase
Chaperone protein	2	Chaperone DnaJ-domain containing protein, Tetratricopeptide repeat domain-containing protein
Ubiquitin-fold modifier	1	Ubiquitin-fold modifier 1
Lignin biosynthesis protein	1	Cinnamoyl-CoA reductase like protein
Transcription factor	2	NAC domain containing protein 47, SCPL20
Stress/defense response	4	Bifunctional Nucleases in basal defense response 1, Bifunctional Nucleases in basal defense response 1, SAP domain-containing protein, putative PAP_fibrillin
Detoxification	5	Putative thiamine biosynthesis protein, putative thiamine biosynthesis protein, putative thiamine biosynthesis protein, thiazole biosynthetic enzyme, *Gossypium hirsutum* alcohol dehydrogenase 1
Photosynthesis	1	Chlororespiratory reduction 3
Metabolism	3	Protein like SEX4 1, phenylalanine ammonia-lyase 1, MAR-binding filament-like protein 1
Electron transport system	1	NDH-dependent cyclic electron flow 1
Transporter	2	Copper transporter 1, nitrate transporter 1.5
Unknown protein	35	Unknown protein
	**Down-regulated transcripts**	
Rector-like protein kinase	1	Protein kinase family protein
Protein phosphatase	1	Serine theronine protein phosphatase PP1
F-box protein	2	EID1-like F-box protein 2, EIN3-binding F-box protein 1
Chaperone protein	1	TCP-1/cpn60 chaperonin family protein
Phosphorylase	1	C2 calcium/lipid-binding and phosphoribosyltransferase C-terminal domain-containing protein
Ribosomal protein	1	60S ribosomal protein L4-1
Post-translational modification	1	Carboxypeptidase
Transcription factor	1	Scarecrow transcription factor
Heat shock protein	2	Heat shock protein cognate 70, HSP20 family protein
Hormone	3	DELLA protein RGA, putative 12-oxophytodienoate reductase, 4-coumarate--CoA ligase-like 7
Stress/defense response	1	Immediate-early salicylate-induced glucosyltransferase
Phototropic response	1	Root phototropism protein 2
Sulfotransferase	1	Flavonol sulfotransferase-like protein
Cell wall biosunthesis	2	Cellulose synthase, pectinesterase 3
Metabolism	6	HXXXD-type acyl-transferase-like protein, putative 3-isopropylmalate dehydratase large subunit, beta-xylosidase 1, glucan endo-1,3-beta-D-glucosidase, OSJNBa0088H09.3, Nitrate reductase (NADH)
Transporter	2	Nitrate transporter 1.4, nitrate transporter 1.4
Other	3	Leucine-rich repeat-containing protein, putative elongation factor 2, putative elongation factor 2
Unknown protein	32	Unknown protein

### Signal transduction

As expected, the genes functional in signal transduction were the most since different abiotic stress share overlapping signal transduction pathways in many steps. Thirteen of 29 commonly induced and 11 from the 30 commonly repressed transcripts took roles in signal transduction and regulation of gene expression under abiotic stress. The first group of genes included kinases, phosphatases, calcium-binding proteins and proteases which were previously shown involved in stress signal transduction. An alpha-glucan phosporylase isozyme H [42] was up-regulated over 20 times by ABA, cold, drought, salinity and pH. We also characterized two alpha-glucan water dikinase 1 [43], and AtPHS2 (alpha-glucan phosporylase), a phosphorylase transferase, all of them were up-regulated over 5 times by these five stress conditions, especially over 10 times by ABA, they were all starch-related and involved in the signal transduction of abiotic stress in *Arabidopsis*. A Cinnamoyl-CoA reductase (CCR-like protein) was found activated in all of the five stress conditions. It was reported that, OsCCR1 is a key enzyme in lignin biosynthesis, and functions as an effector of the small GTPase Rac in defense response in rice [44]. Protein phosphatase (PP) is a type of phosphoprotein cascade, with the function to inactivate the phosphoprotein. There are four subunits in PP, and each one has different interaction partners, while PP2B and PP2C are Ca^2+^-dependent. In our results, we found two well-known ABA signal transduction components, PP2C 77 and PP2C 12, were commonly up-regulated [45,46]. EID1 (Empfindlicher im Dunkelroten Licht 1) is an F-box protein that functions as a negative regulator in phytochrome A (phyA)-specific light signaling [47,48]. Ethylene insensitive 3 (EIN3) is a key transcription regulator mediating ethylene signaling [49,50], EBF1 (EIN3-binding F-box protein 1) and EBF2 interact with EIN3 directly for the ubiquitination and proteolysis of EIN3 [51]. These genes are ubiquitin-protein related but play an important role in the signal transduction of cotton abiotic stress [33]. Serine threonine protein phosphatase PP1 is a highly regulated family of serine/threonine phosphatases required in cell growth and signaling [52,53]. The immediate-early salicylate-induced glucosyl transferase was important not only in biotrophic stress but also for wounding stress [54]. Down-regulation of these genes suggested the crosstalk of stress response in above pathways. 

In addition to the stress signal transduction cascades, the second group featured as genes functioning as cofactors. A Chaperone DnaJ-domain containing protein (Genebank ID: AI727487) [55], a Tetratricopeptide repeat (TPR) protein [56,57] and a ubiquitin-fold modifier 1 (Ufm1) [58] were also identified as commonly up-regulated members, these proteins do not transduce signals directly but work as cofactors in stress tolerance, developmental process, or ER stress. A heat shock protein cognate 70 (Hsp70) [59], a member of small heat shock protein or HSP20 family [60] and a TCP-1 (T-complex protein 1) /cpn60 chaperone family protein [61] were repressed by all five stresses, it means that these proteins were likely involved in stress response, besides their roles in immune sensing, hypersensitive response or protein folding. The 60S ribosomal protein L4A can be directly regulated by auxin and the exogenous application of auxin results in vacuolar trafﬁcking defects of plants [62,63]. The carboxypeptidase is functional in post-translational modification [64,65] and the leucine-rich repeat-containing protein are involved in the formation of protein-protein interaction and signaling [66]. 

Stress response of plant is often mediated by phytohormones such as ABA, ethylene (ET), salicylic acid (SA), jasmonic acid (JA) and gibberellin (GA), it is believed that there is crosstalk between stress adaption/response and hormone regulation pathways, although the hormones may participate in stress reaction independently, synergistically or antagonistically [67]. Consistently, we found that many genes involved in the metabolism of GA and JA were repressed by all five stress conditions. These genes were DELLA protein RGA, 4-coumarate--CoA ligase-like 7 and putative 12-oxophytodienoate reductase [68-70].

### Transcription factor

TFs function as key modular players in stress perception. In our results, two TFs were identified as commonly up-regulated, they were NAC domain containing protein 47 [71], and SCPL 20, a serine carboxypeptidase with a NAC domain that active in the regulation of wood formation in poplar and defense responses against biotic and oxidative stress in rice [72]. A scarecrow (SCR) TF (Protein ID: NP_190990.1) was found commonly repressed [73], it suggested that NAC TF family maybe a kind of common factor that regulated the downstream genes to improve the adaption to various abiotic stresses.

### Stress response/defense

The third portion covered the functional proteins that might take roles in stress tolerance. Bifunctional Nucleases (BFNs) have both RNase and DNase activities. Two BFNs in basal defense response 1 (BBD1) from *Oryza minuta* and *Arabidopsis*, OmBBD1 and AtBBD1 respectively, were investigated recently, and it was reported that these two proteins took regulatory roles in ABA-mediated callose deposition to set up an initial defense barrier against pathogenesis [74]. Interestingly, we found 2 BBD1 proteins from our microarray data, it suggests the existence of shared defensing pathways and transduction signals to abiotic and biotic stresses in plant. There were three putative thiamine biosynthesis proteins induced by the above stresses (Table 1), vitamin B1 (thiamine) is a player during plant adaptation to stresses, mainly oxidative stress [75,76]. Our results also showed that chlororespiratory reduction 3 (CRR3) [77], thiazole biosynthetic enzyme, the precursor of vitamin B1[78], *G. hirsutum* alcohol dehydrogenase 1 (ADH1) [79], PAP (Plastid-lipid associated protein) fibrillin, MAR-binding filament-like protein 1 (MFP1) [80], NDH-dependent cyclic electron flow 1, protein like Starch Excess 4 (SEX4) 1 [81], SAP domain-containing protein, phenylalanine ammonia-lyase 1 (PAL1) were commonly up-regulated.

### Metabolism

 Many genes involved in cell wall metabolism were repressed by all five stress conditions, such as beta-xylosidase 1 (BXL1) [82], cellulose synthase, pectinesterase 3 (PME3) [83], HXXXD-type acyl-transferase-like protein [84] and putative glucan endo-1 and 3-beta-D-glucosidase[85], they were involved in cell wall modification, cell division or growth .

### Transporter facilitation

 There were several genes encoding transporters, such as a copper transporter 1 (COPT1) [86] and a nitrate transporter (NRT) 1.5 [87], were commonly up-regulated, while two NRT 1.4 were commonly down-regulated. Transporters play an important role in ion homeostasis and detoxification of toxic metal ions, no report about their roles in stress response/defense has been found previously, so it is worthy of further investigation. 

### Quantitative real-time PCR validation

**Figure 4:**
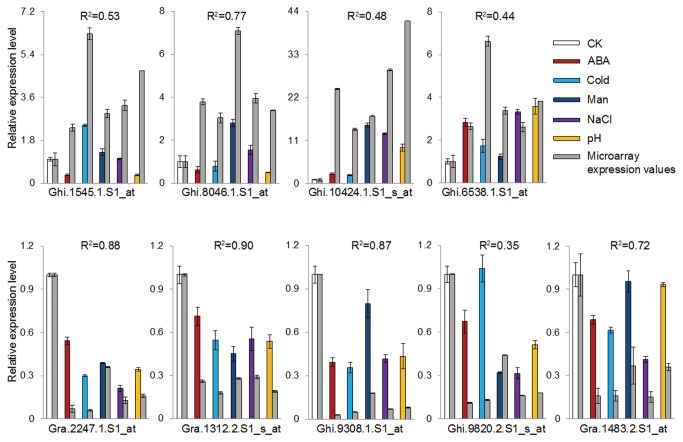
Quantitative real-time PCR validation for selected transcripts. The columns indicate the qRT-PCR values of CK (white bar), ABA (red bar), Cold (light blue bar), Man (dark blue bar), NaCl (purple bar) and pH (yellow bar), respectively. The columns (grey bar) indicate the relative microarray expression value of crossponding stress treatment. CK, control plants; pH, pH=11; ABA, 1μM ABA; Man, 200mM mannitol; Cold, 4°C; NaCl, 200mM NaCl.

In order to validate differentially expressed genes identified from microarray analysis, we repeated the stress treatments and isolated RNA from samples. Nine interesting genes that were commonly regulated under all of the five stresses were selected for validation with qRT-PCR. Four commonly induced transcripts for CRR3 (Ghi.1545.1.S1_at), GhADH1 (Ghi.8046.1.S1_at), putative thiamine biosynthesis protein (Ghi.10424.1.S1_s_at), NAC domain containing protein (Ghi.6538.1.S1_at) and five commonly repressed transcripts encoding NRT1.4 (Gra.2247.1.S1_at), SCR TF (Protein ID: NP_190990.1) (Gra.1312.2.S1_s_at), a HSP20 family protein (Ghi.9308.1.S1_at), immediate-early salicylate-induced glucosyltransferase (Ghi.9820.2.S1_s_at), pectinesterase 3 (Gra.1483.2.S1_at) were verified with qRT-PCR (Figure 4, Table S5). Comparison of the results from qRT-PCR analysis revealed the consistent expression pattern with our microarray data, and the Person’s correlation coefficient between the two sets of data was between 0.4 and 0.9 (Figure 4, Table S5).

### TFs responsible to ABA, cold, drought, salinity and pH stress

There are a large number of TFs encoded by plant genome to perceive and mediate response to environmental changes. These TFs act as the earliest and vital players during stresses [88]. Genome-wide transcriptome analysis revealed that a number of TFs were induced or repressed in response to diverse abiotic stresses. Among the genes induced by ABA, cold, drought, salinity or pH, we found several TF families, suggesting that various transcriptional regulatory mechanisms were active in stress signal transduction pathways (Table 2, Table S6). Stress-activated or repressed TF genes were identified from 10 different families including WRKY, NAC, MYB, AP2-ethylene (AP2/ERF), and C2H2 like zinc finger, auxin response factor (ARF), G-box, bHLH, bZIP, and TCP. In ABA stress network, 15 WRKY genes were induced, 6 ethylene genes were repressed, corresponding to 19% and 21.4% of all ABA-induced and repressed TFs respectively. For cold stress response, 14 AP2-ethylene and 13 C2H2 like zinc finger genes were induced, 4 MYB genes and 3 bZIP genes were repressed, taking 50% and 24.1% of all cold-induced and repressed TFs. There are 18 AP2-ethylene genes, 17 WRKY genes and 11 NAC genes were induced by drought, corresponding to 47.4% of all drought-induced TFs. Three ERF genes, 3 C2H2 like zinc finger genes and 3 bZIP genes were repressed by salinity treatment, covering 90% of all salinity-repressed TFs, whereas under pH stress treatment, 19 WRKY genes, 16 AP2-ethylene genes and 10 C2H2 like zinc finger genes were induced, 4 C2H2 like zinc finger genes and 3 ERF genes were repressed, proportioning 36.1% and 30.4% of all pH-induced and repressed TFs respectively. TFs are important regulators primarily involved in the initiation stage of RNA transcription. Recently, several reports have shown up-regulation of these TF families in response to biotic stresses in cotton, and DREB-binding TF gene and ERF TF gene were isolated and identified [31,33,89-91]. According to our results, WRKY family was the most highly expressed TF , while ERF was the most important repressed TF that mediating transcription of stress-responsible genes under various abiotic stress conditions, and this was consistent with the repressed genes EID1 and EIN3 observed in the commonly inhibited genes, they are the important components of ethylene signaling pathway of stress response [32].These results also suggested that both ABA-dependent and -independent pathways were induced under various stress. However, we only identified 2 transcription factors that were common to all of the 5 kinds of abiotic stress, while more than 30 transcription factors were common to ABA, drought, cold and alkalinity stress (Table S6), this suggested that there was possibly a TF network to regulate various stresses including drought, cold and alkalinity, and pathways for different stresses shared cross-talk at many steps. We did not find much TFs to salinity stress, this was likely due to cotton was more adaptable to salinity than other plants, so that the anti-salinity genes were always on in cotton and not so many TFs were necessary for salinity tolerance or adaption.

**Table 2:** Summary of differentially expressed transcription factors (TFs) under ABA, cold, drought, high-salinity and pH stress conditions.

**TF family**	**Up-Regulated Transcripts**									
	**ABA**	**%**	**Cold**	**%**	**Mannitol**	**%**	**NaCl**	**%**	**pH**	**%**
WRKY	15	19.0	10	18.5	17	17.5	0	0	19	19.6
NAC	1	1.3	0	0	11	11.3	1	12.5	2	2.1
MYB	6	7.6	6	11.1	8	8.2	1	12.5	8	8.2
AP2-ERF	6	7.6	14	25.9	18	18.6	0	0	16	16.5
C2H2 like Zinc finger	5	6.3	13	24.1	0	0	1	12.5	10	10.3
Auxin response factor	5	6.3	0	0	3	3.1	1	12.5	2	2.1
G-box	3	3.8	2	3.7	2	2.1	1	12.5	3	3.1
bHLH	4	5.1	1	1.9	3	3.1	2	25.0	3	3.1
bZIP	5	6.3	0	0	6	6.2	0	0	4	4.1
TCP	2	2.5	0	0	0	0	0	0	0	0
Other	27	34.2	8	14.8	29	29.9	1	12.5	30	30.9
Total	79	100	54	100	97	100	8	100	97	100
**TF family**	**Down-Regulated Transcripts**									
	**ABA**	**%**	**Cold**	**%**	**Mannitol**	**%**	**NaCl**	**%**	**pH**	**%**
WRKY	1	3.6	0	0	1	6.25	0	0	0	0
NAC	2	7.1	1	3.4	0	0	0	0	1	4.3
MYB	2	7.1	4	13.8	1	6.25	0	0	2	8.7
ERF	6	21.4	0	0	1	6.25	3	30.0	3	13.0
C2H2 like Zinc finger	3	10.7	1	3.4	1	6.25	3	30.0	4	17.4
Auxin response factor	0	0	0	0	0	0	0	0	1	4.3
G-box	1	3.6	0	0	0	0	0	0	0	0
bZIP	1	3.6	3	10.3	1	6.25	3	30.0	2	8.7
bHLH	0	0	2	6.9	0	0	0	0	0	0
TCP	1	3.6	1	3.4	1	6.25	0	0	0	0
Other	11	39.3	17	58.6	10	62.5	1	10.0	10	43.5
Total	28	100	29	100	16	100	10	100	23	100

Percentage refers to the ratio of transcripts of each TF family relative to total up-regulated or down-regulated TFs identified in the microarray data.

## Conclusion

In this study, we identified 3242, 1231, 3179, 480, and 3378 genes induced by ABA, cold, drought, salinity and pH stress, respectively. We further found out 64 or 62 common genes were induced or repressed by these five kinds of stresses simultaneously. Our results indicated that, comparative microarray analysis was a powerful tool for the identification of multiple stress-inducible genes. These results highlighted the existence of crosstalk among ABA, cold, drought, salinity and pH stresses on plants. Most of the commonly changed genes were involved in signal transduction, stress response/defense and metabolism. WRKY family was implicated as a major type of TF involved in the response to ABA, cold, drought, salinity and pH stress of cotton. However, we noticed that the function of a number of these genes still remained unknown. The functional study on stress involved genes is demanded badly, not only for further understanding of the mechanisms underlying stress response and tolerance of plants, but also improving the stress adaption of crops such as cotton by genetic manipulation. 

Based on our microarray results, we will further investigate the function of key genes involved in plant-stress interaction possibly by reverse genetic method. It is no doubt that the transciptome profiling should benefit the isolation of candidate genes, functional analysis, promoter sequences identification, molecular breeding and genetic manipulation of crops. 

## Materials and Methods

### Plant materials and stress treatments

An Upland cotton (*Gossypium hirsutum* L.), C312 (Coker 312) was used in this study at Institute of Genetics and Developmental Biology, Chinese Academy of Sciences (IGDB, CAS). Healthy seeds of cotton were surfacely sterilized with 75% ethanol for 1 minute, and soaked in 10% H_2_O_2_ for 2 hours, followed by washing thoroughly with sterile water, then kept in double distilled water overnight. 

The sterilized seeds were germinated and grown on half strength MS solid medium (Murashige and Skoog medium) [92] for 4 days in a growth chamber of 25°C, 12h photoperiod (white fluorescent light at 500–600 μm m^-2^s^-1^). For abiotic stress treatment, the seeds were raised on the corresponding medium: for ABA treatment, seeds were kept in 1/2 MS+1μM ABA, pH 5.8 for ten days; for cold treatment, seeds were first kept on 1/2 MS, pH 5.8, and 25°C for 8 days, then exposed to 4°C for 2 days; for drought stress, seeds were treated with 200mM mannitol, pH 5.8, and 25°C for 10 days; for salinity stress, seeds were treated with 200mM NaCl, pH 5.8, and 25°C for 10 days; for alkalinity stress (high pH, hereinafter referred to “pH stress”), seeds were kept in 1/2 MS, pH 11, and 25°C for 10 days. The control and stressed plants were used for RNA extraction and the subsequent microarray experiment.

### RNA isolation

The seedlings of 14-day after germination were harvested and immediately frozen in liquid nitrogen. A modified LiCl method [93] was used for RNA isolation. The total RNA was checked with a NanoDrop ND-1000 spectrophotometer followed by gel electrophoresis, while the intactness of RNA was verified by Agilent 2100 Bioanalyzer.

### Microarray hybridization and data analysis

For the microarray experiment, RNA was pooled from the three biological replicates based on each abiotic treatment. Cleaned total RNAs from each of the six samples, including control, ABA, cold, Mannitol, NaCl as well as pH stress were sent to CapitalBio Corporation in Beijing for microarray hybridization and preliminary data analysis. Affymetrix GeneChip^®^ Cotton Genome Array with 23, 977 probes on the chip were used for hybridization. The arrays of specimens were hybridized with Cy3 and Cy5 labeled probe pairs of ABA treated plants plus control, cold-treated plants plus control, mannitol-treated plants plus control, NaCl-treated plants plus control, and pH-treated plants plus control. To assess the reproducibility of the microarray analysis, we repeated the same experiment three times. Data analysis was performed using SAM&R software. Genes with Fold Change ≥ 2 and FDR (False Discovery Rate) (%) ≤ 5 were identified as differentially expressed genes [94,95]. The analysis of the quality and the reproducibility among the experiments were performed according to the Affymetrix Statistical Algorithms Description Document (2002) by Affymetrix. The microarray data were deposited at GEO (Gene Expression Omnibus) at the National Center for Biotechnology Information (NCBI) http://www.ncbi.nlm.nih.gov/geo/ with the accession number GSE50770.

### qRT-PCR

Nine common differentially expressed genes were selected for real-time quantitative RT-PCR based on the microarray results. A cotton *Actin 7* (GenBank ID: DQ402078.1) gene was used as a standard control in the qRT-PCR reactions. The specificity of all primers was checked via BLASTN searches (http://blast.ncbi.nlm.nih.gov/Blast.cgi). A two-step RT-PCR procedure was performed in all experiments. The first-strand cDNAs were synthesized in a 20 µL reaction solution containing 2μg total RNA samples of control plants, as well as abiotic stressed plants using PrimeScript^®^ RT reagent kit with gDNA Eraser (Perfect Real Time) Kit (TaKaRa, Japan, Catalog No. DRR047A). The real-time amplification reactions were performed using the iCycler iQ thermocycler (Bio-Rad) and the SuperReal PreMix (SYBR Green) Kit (Tiangen, Beijing, Catalog No. FP204) followed the manuals from the manufacturers. The relative value for expression level of each gene was calculated by the equation Y=2^ΔCt^ (ΔCt is the differences of Ct between the control GhActin 7 products and the target gene products). 

### Accession

The microarray data of this report were deposited at GEO (Gene Expression Omnibus) at the National Center for Biotechnology Information (NCBI) http://www.ncbi.nlm.nih.gov/geo/ with the accession number GSE50770.

## Supporting Information

Tables S1 **Cotton microarray annotation data.** (XLSX)

Tables S2 **Cotton microarray GO analysis.** (XLSX)

Tables S3 **Cotton microarray Venn diagram data.** (XLSX)

Tables S4 **Commonly changed transcripts under different treatment.** (XLSX)

Tables S5 **QRT-PCR primer information.** (XLSX)

Tables S6 **Transcription factors under different treatment.** (XLSX)
